# Functional, genetic and bioinformatic characterization of a calcium/calmodulin kinase gene in *Sporothrix schenckii*

**DOI:** 10.1186/1471-2180-7-107

**Published:** 2007-11-29

**Authors:** Liz Valle-Aviles, Shirley Valentin-Berrios, Ricardo R Gonzalez-Mendez, Nuri Rodriguez-del Valle

**Affiliations:** 1 Department of Microbiology and Medical Zoology, Medical Sciences Campus, University of Puerto Rico, PO Box 365067, San Juan, PR 00936-5067, USA; 2 Department of Microbiology, San Juan Bautista School of Medicine, Box 4968, Caguas, PR 00726-4968, USA; 3 Department of Radiological Sciences, Medical Sciences Campus, University of Puerto Rico, PO Box 365067, San Juan, PR 00936-5067, USA

## Abstract

**Background:**

*Sporothrix schenckii *is a pathogenic, dimorphic fungus, the etiological agent of sporotrichosis, a subcutaneous lymphatic mycosis. Dimorphism in *S. schenckii *responds to second messengers such as cAMP and calcium, suggesting the possible involvement of a calcium/calmodulin kinase in its regulation. In this study we describe a novel calcium/calmodulin-dependent protein kinase gene in *S. schenckii, sscmk1*, and the effects of inhibitors of calmodulin and calcium/calmodulin kinases on the yeast to mycelium transition and the yeast cell cycle.

**Results:**

Using the PCR homology approach a new member of the calcium/calmodulin kinase family, SSCMK1, was identified in this fungus. The cDNA sequence of *sscmk1 *revealed an open reading frame of 1,221 nucleotides encoding a 407 amino acid protein with a predicted molecular weight of 45.6 kDa. The genomic sequence of *sscmk1 *revealed the same ORF interrupted by five introns. Bioinformatic analyses of SSCMK1 showed that this protein had the distinctive features that characterize a calcium/calmodulin protein kinase: a serine/threonine protein kinase domain and a calmodulin-binding domain. When compared to homologues from seven species of filamentous fungi, SSCMK1 showed substantial similarities, except for a large and highly variable region that encompasses positions 330 – 380 of the multiple sequence alignment. Inhibition studies using calmodulin inhibitor W-7, and calcium/calmodulin kinase inhibitors, KN-62 and lavendustin C, were found to inhibit budding by cells induced to re-enter the yeast cell cycle and to favor the yeast to mycelium transition.

**Conclusion:**

This study constitutes the first evidence of the presence of a calcium/calmodulin kinase-encoding gene in *S. schenckii *and its possible involvement as an effector of dimorphism in this fungus. These results suggest that a calcium/calmodulin dependent signaling pathway could be involved in the regulation of dimorphism in this fungus. The results suggest that the calcium/calmodulin kinases of yeasts are evolutionarily distinct from those in filamentous fungi.

## Background

*Sporothrix schenckii*, the etiologic agent of sporotrichosis, is a dimorphic fungus that produces lymphocutaneous lesions [1]. Pathogenic fungi use signal transduction pathways to rapidly adapt to changing environmental conditions. Studies on the molecular and cellular events during the dimorphic transitions of *S. schenckii *suggested a role for calcium ions in the control of proliferation and morphogenesis in this fungus [2]. Studies on the role of calcium metabolism during germ tube formation in *S. schenckii *yeast cells showed that extracellular calcium ions stimulate the yeast to mycelium transition and that two calcium uptake peaks were detected in cells undergoing transition from the yeast to mycelium forms [3]. The first calcium uptake peak occurred during the first 30 min after the induction of the yeast to mycelium transition. The second calcium uptake peak was observed 300 min after induction, at the time of DNA synthesis. When different substances that affected calcium uptake were added to the medium during the yeast to mycelium transition such as cobalt ions, ionophore A23187 and compound R24571, germ tube formation was inhibited or occurred with reduced kinetics [3].

Calcium is one of the most important intracellular second messengers, it is involved in a wide range of cellular events including secretion, motility, intermediary metabolism, ion channel activity, and gene expression [4-8]. An increase in intracellular calcium concentration results from any of two events: the release of calcium from internal stores or the increased uptake from the extracellular environment. Once the intracellular calcium concentration has increased, calcium exerts its role through a specific class of proteins known as calcium binding proteins. One of the most of important of these proteins is calmodulin (CaM) [9,10]. Among the CaM interacting proteins are the Ca^2+^/calmodulin-dependent protein kinases (CaMKs) [11-14].

The members of the CaMK family are usually classified based on their substrate specificity into two major groups. The first group has a broad substrate specificity characterized by the ability to phosphorylate many different proteins and includes CaMKs I, II and IV. Within this group, CaMKs I and IV are monomeric enzymes while CaMK II is a multimeric enzyme. The second group is characterized by narrow substrate specificity, and includes phosphorylase kinase, myosin light chain kinase and CaMK III (eEF-2 Kinase) [15].

Calcium/calmodulin kinases are serine/threonine protein kinases. They share many common structural features, having two major domains: an amino-terminal catalytic domain that is highly conserved, and a carboxy-terminal regulatory domain. The regulatory domain consists of overlapping autoinhibitory and Ca^2+^/CaM binding domains. The autoinhibitory domain acts as a pseudosubstrate, mimicking the substrate and interacting with the catalytic domain, blocking access of the true substrate to the catalytic site [15,16]. The Ca^2+^/CaM binding domain is located in the C-terminal portion of the enzyme, consisting of approximately 20 amino acids. Upon binding of Ca^2+^/calmodulin to a CaM-binding domain in the regulatory domain of the CaMK, a conformational change ensues in which the autoinhibitory domain is removed from the catalytic domain, exposing the active site of the kinase and enabling binding of the substrate and its subsequent phosphorylation [10,15]. Activation of CaMK I and IV is also regulated by phosphorylation by a CaM kinase kinase (CaMKK) [16].

Within this group of multifunctional Ca^2+^/calmodulin kinases, the most studied has been the multimeric CaMK II [12,15]. Very little has been published concerning CaMK I [17]. This monomeric enzyme is primarily cytoplasmic in mammalian cells and those of many other organisms [17]. Multiple isoforms of CaMK I have been identified that have either cytoplasmic and/or nuclear localization. A number of substrates have been identified for CaMK I including synapsin I and II, the cystic fibrosis transmembrane conductance regulator, myosin II regulatory light chain kinase, cAMP response element binding protein (CREB), activating transcription factor (ATF-1), histone deacetylases 4 and 5 and translation initiator factor eIF4GII [18].

The role of CaMKs in mammalian systems, particularly in neurons is well established [11], while their occurrence and function in fungi is not fully documented. Pausch and colleagues cloned two Ca^2+^/calmodulin-dependent protein kinases, CMK1 and CMK2, from *Saccharomyces cerevisiae *[19]. The *cmk1 *gene encodes a product of approximately 55 kDa. Several functions of CaMKs in *S. cerevisiae *include: survival of pheromone-induced growth arrest, salt tolerance and thermotolerance [20]. Rasmussen reported the presence of a Ca^2+^/calmodulin-dependent protein kinase gene in the yeast *Schizosaccharomyces pombe, cmk1*, with 40% similarity to the rat and human CaMK-I [21]. In the filamentous fungus *Aspergillus nidulans*, three calcium/calmodulin dependent protein kinases, CMKA, CMKB, and CMKC, have been reported [22-24]. CMKA and CMKB were identified as CaMKs while CMKC was identified as a CaMKK. The disruption of the CMKA and CMKB encoding genes was reported to be lethal [25]. In another filamentous fungus, *Neurospora crassa*, a Ca^2+^/calmodulin-dependent protein kinase (CaMK-I) was reported by Yang and collaborators [26]. This protein was 71% similar to Ca^2+^/calmodulin dependent protein kinase A of *A. nidulans *and 53% similar to CaMK-II of *S. cerevisiae*. Tsai, Tu and Chen described a Ca^2+^/calmodulin-dependent protein kinase gene in the filamentous fungus, *Arthrobotry*s *dactyloides *[27]. This gene encoded a protein with significant homology to mammalian CaMKs. Whole genome sequencing projects show the presence of hypothetical proteins homologous to CaMK in other fungi, such as *Magnaporthe grisea *and *Gibberella zeae *although the formal identification of these proteins has not been reported in the scientific literature.

Even though the importance of calcium signaling in the control of dimorphism in *S. schenckii *is well documented, the presence of key enzymes involved in calcium signaling in this fungus have not been described [2,3]. The experimental objectives of this work were to establish the presence of a Ca^2+^/calmodulin-dependent protein kinase gene in *S. schenckii *using a PCR homology approach and the characterization of the role of the encoded kinase in *S. schenckii *dimorphism. We present details of the identification and sequencing of the *sscmk1 *gene, gene organization, including the presence and position of introns, and conserved polypeptide-encoded domains. We establish the relationship of this gene to those of other filamentous fungi using bioinformatics and phylogenetic analyses and present evidence for the existence of a well-defined group of CaM kinases in the filamentous fungi. We establish the expression of this gene in the yeast and mycelium forms of *S. schenckii*. We also inquire into the effects of compound W-7, a calmodulin inhibitor, and KN-62 and lavendustin C, CaMK inhibitors, on the yeast to mycelium transition and the yeast cell cycle in *S. schenckii*. This work constitutes the first direct evidence of the presence of a CaMK encoding gene in *S. schenckii *and its possible involvement on dimorphism in this fungus. These results suggest that a calcium/calmodulin dependent signaling pathway could be involved in the regulation of dimorphism in this fungus and will ultimately help us elucidate the calcium dependent signal transduction pathways in *S. schenckii*.

## Results

In this study we describe a calcium/calmodulin-dependent protein kinase gene in *S. schenckii, sscmk1*, and the effects of inhibitors of calmodulin and calcium/calmodulin kinases on the yeast to mycelium transition and the yeast cell cycle.

### Identification and characterization of the *sscmk1 *gene

The PCR homology approach was used to identify the presence of a calcium/calmodulin-dependent kinase gene in *S. schenckii *DNA with primers based on conserved motifs of the *camk-1 *gene of *N. crassa *[26]. Several sets of primers were tested. A 363 bp PCR product was obtained using *S. schenckii *DNA as template with an upper primer comprising nucleotides 352 to 369 and as lower primer the antisense sequence included between nucleotides 610 to 627 in the coding region of the *N. crassa camk-1 *gene. This PCR product contained 275 bp of coding sequence and 88 bp of a putative intron sequence, and included the sequence from nucleotides 653 to 1016 (5'->3') of the *S. schenckii *Ca^2+^/CaM-dependent protein kinase genomic sequence. The derived amino acid sequence of this PCR product was found to be 91% identical to the corresponding region of the *N. crassa camk-1*. This PCR product was the first evidence of the presence of a calcium/calmodulin-dependent protein kinase gene in *S. schenckii *and was named *sscmk1*.

Based on the sequence of the original 363 bp PCR product, gene-specific primers and nested gene-specific primers were designed for RACE as described in Methods. The 5' and 3' ends of the *sscmk1 *gene were obtained from two different RACE reactions each.

**Figure 1:**
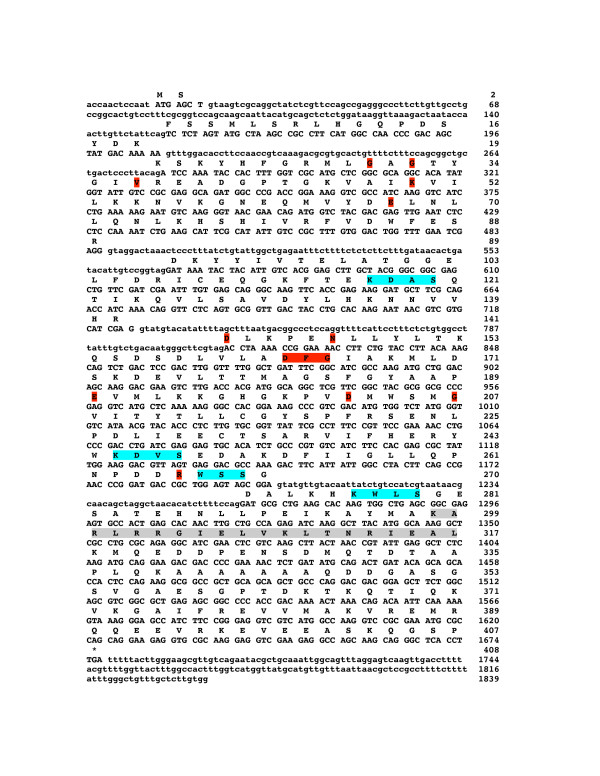
**Genomic and derived amino acid sequences of the *S. schenckii sscmk1 *gene**. The nucleotide sequence of the *sscmk1 *gene is shown. Non-coding regions are given in lower case letters, coding regions and amino acids are given in upper case letters. The derived amino acid sequence shows a protein of 407 amino acids. The invariant amino acids required by serine/threonine protein kinases are shaded in red. The potential autophosphorylation sites containing the consensus sequence {R, K}-X-X-{T, S} are shaded in light blue. The calmodulin-binding domain is shaded in gray.

PCR reactions were performed to confirm both the genomic and cDNA sequences of the *sscmk1 *gene. The genomic DNA PCR product was 1839 bp, showing the presence of an open reading frame (ORF) of 1221 bp interrupted by five introns (Figure 1). The coding sequence in this PCR product confirmed the one obtained using the RACE technique. The introns had the consensus intron/exon junction splice site, 5'GT/3'AG. The position of the introns in the *sscmk1 *genomic sequence were the following: the first intron had 136 bp and included nucleotides 20 to 155 (5'->3'); the second intron had 71 bp and included nucleotides 208 to 278 (5'->3'); the third intron had 82 bp and included nucleotides 487 to 568 (5'->3'); the fourth intron had 88 bp and included nucleotides 726 to 813 (5'->3') and the fifth intron had 64 bp and included nucleotides 1200 to 1263 (5'->3') (Figure 1).

The position of the introns in *sscmk1 *was conserved when compared to those of the introns in the *camk-1 *gene of *N. crassa*, although the length and the nucleotide sequence of the respective introns were not the same. The first and fourth introns are phase 1 introns and were found interrupting codons encoding F3 and D142, respectively. The second intron was a phase 2 intron and was found interrupting the codon that encoded K20. The third and fifth introns in *sscmk1 *are phase 0 introns, interrupting the coding region between the codons encoding R89 and D90, G270 and D271, respectively.

The cDNA coding sequence was obtained using *S. schenckii *yeast cDNA as template. A PCR product of 1790 bp was obtained, containing a 1221 bp coding sequence, 378 nucleotides of the 5' UTR and 162 nucleotides of 3' UTR after the termination codon prior to the poly A+ tail. The coding sequence was exactly the same as the previous sequence obtained using genomic DNA as template, except for the absence of the intron sequences (Figure 1).

**Figure 2:**
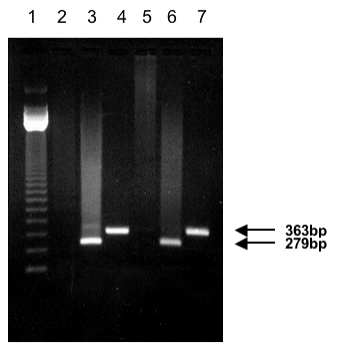
**RT-PCR using *S. schenckii *yeast and mycelium RNA of a conserved region in *sscmk1 *gene**. Total RNA was extracted from yeast and mycelium cells of *S. schenckii *and used as templates for RT-PCR using the DASQTI/WSMGVI primer pair. Fifteen μl of each reaction were resolved in a 1.2% agarose gel electrophoresis. Lanes 3 and 6, show the results obtained from the RT-PCR using total RNA extracted from yeast and mycelium cells respectively, and show the 279 bp band. Lanes 2 and 5 represent the RT-PCR control for each time point where no M-MLV reverse transcriptase was added. Lanes 4 and 7 show a 363 bp product corresponding to a control PCR reaction using genomic DNA as template and the same primer pair. Lane 1 shows the 123 bp DNA Ladder. The position of each of the RT-PCR products is indicated by arrows.

In order to determine if this *sscmk1 *gene was transcribed in the mycelium form of the fungus, total RNA from the mycelium and yeast forms of the fungus were used to synthesize cDNA that was used as template for RT-PCR reactions with the DASQTI/WSMGVI primer combination. This set of primers amplified the same region obtained in the original 363 bp PCR product without the intron sequence of 88 bp. Figure 2, shows the expected PCR product of 279 bp obtained when using yeast and mycelium cDNA as template demonstrating the presence of the *sscmk1 *gene transcript in both forms of the fungus.

### Bioinformatic sequence characterization

**Figure 3:**
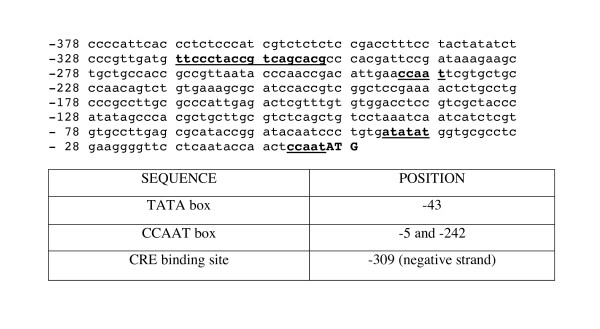
**Analysis of the partial 5' UTR sequence of the *sscmk1 *gene cDNA**. Predicted regulatory element and binding sites for the partial 5' UTR of the cDNA sequence of the *sscmk1 *are shown in bold and underlined, and the type and location are shown.

Figure 3 shows the 5' UTR of the cDNA sequence that was analyzed using MatInspector [28] with 85% as threshold, and identified the presence of a TATA box at position -43. The position of this TATA box is within the region we would expect to find it (between -25 to -100) in reference to the initial methionine. The presence of two CCAAT boxes at -5 and -242 was also observed. A putative CRE biding site was observed at -309. It is of interest to note that CaMKs in many systems are responsible, together with cAMP for the activation of the transcription factor that binds to CRE [29], if this applies to our CaMK, it could be modulating its own transcription by this mechanism.

Figure 1 shows the derived amino acid sequence of *sscmk1*, a protein of 407 amino acids with a calculated molecular weight of 45.6 kDa. Protein domain analysis of SSCMK1 using the online ScanProsite motif search tool [30] and Pfam [31], identified the presence of a protein kinase ATP-binding region from amino acids L29 to K54 and a serine/threonine protein kinase active site in this new sequence between amino acid residues K22 and L278. All residues required for ATP binding and kinase activity are conserved in the amino acid sequence derived from *sscmk1 *together with the predicted calmodulin-binding site, suggesting that this new gene encodes a Ca^2+^/CaMK. The 12 highly conserved regions referred to as subdomains characteristic of protein kinases by Hanks and collaborators [32,33] and the calcium/calmodulin binding domain are also present in this protein.

Using the PANTHER Classification System the *sscmk1 *gene was classified as a member of the calcium/calmodulin-dependent protein kinase subfamily (PTHR22982:SF29) (residues 67–342) with an E-value of 1.8 × e^-187 ^indicating a very likely correct functional assignment [34]. According to the PANTHER ontologies, the molecular function is that of a non-receptor serine/threonine protein kinase and *sscmk1 *is involved in molecular processes involving protein phosphorylation and calcium mediated intracellular signaling.

The putative CaM-binding domain was also identified in SSCMK1 using the Calmodulin Target Database [35]. The Calmodulin Target Database search identified the presence of a 1-5-8-14 CaM-binding motif at residues 298 to 317, (KARLRRGIELVKLTNRIEAL).

Further analysis of the *sscmk1 *gene encoded product showed a pak-box/p21-rho-binding domain. The Pfam search identified Rho binding repeats at amino acids 97–150 and 185–234 with e values of 1.6e^-06 ^and 4.9e^-07^, respectively.

**Figure 4:**
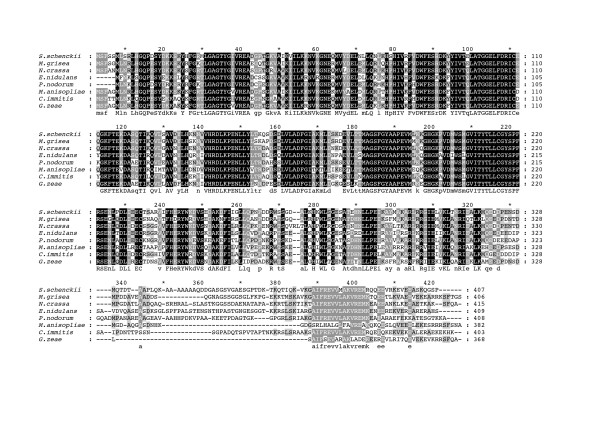
**Multiple sequence alignment of the calcium/calmodulin-dependent protein kinases from filamentous fungi**. In the alignment, black shading with white letters indicates 100% identity, gray shading with white letters indicates 75–99% identity, gray shading with black letters indicates 50–74% identity. The Ca^2+^/calmodulin binding domain is located on positions 300–319 of the alignment. The serine/threonine protein kinase domain is located between positions 22 and 280 of the alignment.

Figure 4 shows a multiple sequence alignment of the derived amino acid sequence of *sscmk1 *to seven other calcium/calmodulin-dependent protein kinases from filamentous fungi. These results show a high degree of conservation among these proteins specifically at the kinase domain. We calculated pairwise identities based on the alignment and found that sequence identity goes from 59% to 74% in these homologues (data not shown). There is substantial variability at the carboxy-terminal of these proteins, which can be seen after the camodulin-binding domain, between positions 330 and 420 of the alignment. All the required conserved residues and domains can be seen in this alignment.

### Phylogenetic analysis

**Figure 5:**
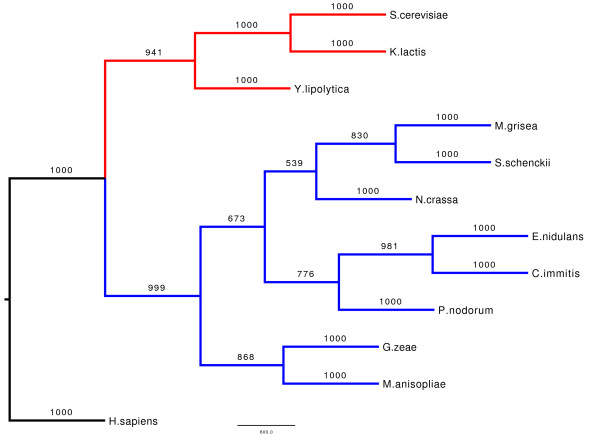
**Phylogenetic tree of calcium/calmodulin-dependent protein kinase homologues from fungi**. Phylogenetic tree of CaMKs from fungi using the human CaMK1D as an outgroup. The yeast CaMKs are shown in red, the filamentous fungi CaMKs are shown in blue. The bootstrap values for each branch are shown (maximum value 1000).

Figure 5 shows the rooted consensus phylogenetic gene tree resulting from the analysis of several CaMKs from fungi using human CaMK1D as an outgroup. The human CaMK1D was chosen based on the results of a BLAST search giving the highest similarity and significance among the human CaMK1s. This analysis identified the *S. schenckii sscmk1 *gene as an orthologue of the CaMKs of the filamentous fungi, and strongly supports the evolution of two groups of these kinases in fungi, those belonging to the yeasts and those belonging to the filamentous fungi. Bootstrap values for the node branching the yeast CaMKs from the filamentous fungi CaMKs are over 90%, indicating an extremely well supported separation between these two groups. The gene tree follows almost exactly the species tree obtained from the NCBI Entrez Taxonomy resource [36,37], indicating that the CaMK1 evolutionary differences are a reflection of the evolution of the species used to construct the tree.

### Effects of CaM and CaMK inhibitors on the yeast to mycelium transition and on the yeast cell cycle

**Figure 6:**
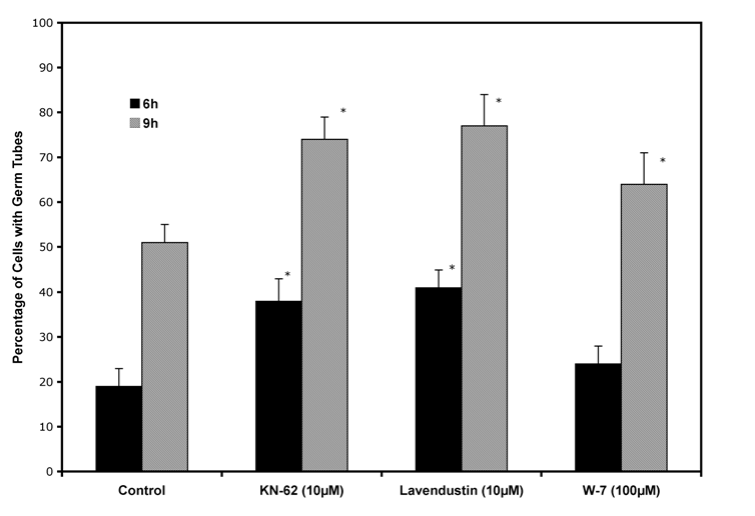
**Effects of CaM and CaMK inhibitors on the yeast to mycelium transition**. Yeast cells grown, harvested and selected by filtration as described in Methods were induced to form germ tubes in a basal medium with glucose at pH 4.0 and incubated at 25°C in the presence and absence of compound W-7, KN-62 and lavendustin C. All values are given as the average percentage ± one SD of for at least three independent experiments. The Student's t test was used to determine the statistical significance of the data at a 95% confidence level. Values that differ significantly from those of the control at 95% confidence level are marked with an asterisk.

Figure 6 shows the percent stimulation of germ tube formation in yeast cells induced to undergo transformation to the mycelium form of the fungus in the presence and absence of the calmodulin inhibitor, compound W-7 [38-40], and the calcium/calmodulin kinase inhibitors, KN-62 [41,42] and lavendustin C [43]. This figure demonstrates that calcium/calmodulin kinase inhibitors significantly stimulated the yeast to mycelium transition at 6 and 9 h of incubation while the stimulation observed with the CaM inhibitor, W-7, was significant at 9 h of incubation. The CaM kinase inhibitors, KN-62 and lavendustin C, showed a stimulation of approximately 100% at 6 h after inoculation. After 9 h of incubation, KN-62 and lavendustin C stimulation of germ tube formation was approximately 50%, while W-7 stimulation was 28%.

**Figure 7:**
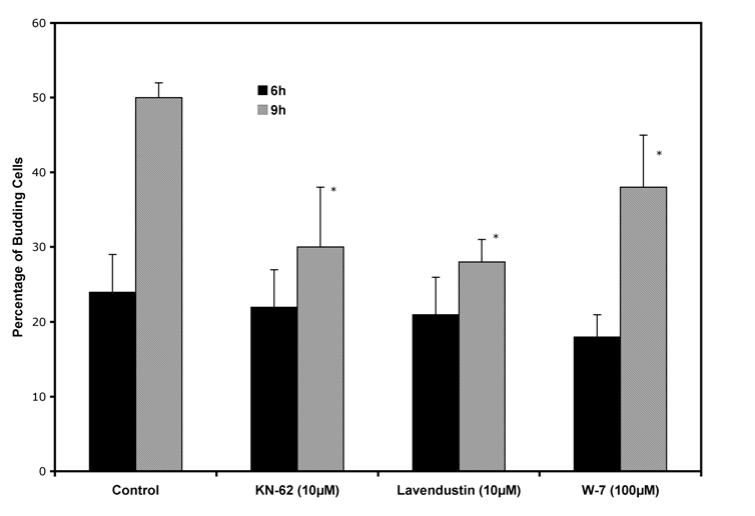
**Effects of CaM and CaMK inhibitors on the yeast budding cycle**. Yeast cells grown, harvested and selected by filtration as described in Methods were induced to re-enter the budding cycle in a basal medium with glucose at pH 7.2 and incubated at 25°C in the presence and absence of W-7, KN-62 and lavendustin C. All values are given as the average percentage ± one SD of for at least three independent experiments. The Student's t test was used to determine the statistical significance of the data at a 95% confidence level. Values that differ significantly from those of the control at 95% confidence level are marked with an asterisk.

Figure 7 shows the percentage of budding in cells induced to re-enter the yeast cell cycle in the presence and absence of calmodulin inhibitor, compound W-7, and calcium/calmodulin kinase inhibitors, KN-62 and lavendustin C. This figure shows no effect of any of these compounds at 6 h after inoculation while after 9 h of incubation all of these compounds significantly inhibited the yeast cell cycle. The inhibition observed after 9 h of incubation was approximately 40% for KN-62 and lavendustin C. For W-7 the inhibition observed after 9 h of incubation was 24%. During the yeast cell cycle, the observed inhibition caused by the addition of W-7 was less than that observed in the presence of inhibitors of CaM kinases.

## Discussion

In this study we describe a calcium/calmodulin kinase gene in *S. schenckii, sscmk1*, and the effects of inhibitors of the encoded protein on the yeast to mycelium transition and the yeast cell cycle. CaM kinases have been extensively recognized as important regulators of morphogenesis and differentiation in many different cell types [11,12]. The present study was conducted in order to establish the presence of this important gene in *S. schenckii *and the potential involvement of CaMK on the control of the dimorphism of this fungus using CaM and CaM kinase inhibitors.

The CaM kinase encoded by the *sscmk1 *gene in *S. schenckii *is homologous to members of the CaMK group in such diverse organisms as *Homo sapiens*, *Xenopus*, *Drosophila *and definitely fungi as shown in Figure 4 and 5. Our sequence and phylogenetic analyses show a very high degree of conservation of this protein in fungi. In particular, that the CaMKs of the yeast and the filamentous fungi show distinct monophyletic groups.

The derived amino acids sequence of the *sscmk1 *gene encodes a protein of 407 amino acids. The number of amino acid residues present in this protein is within the range of other fungal CaMKs, such as: 415 amino acids in *N. crassa*, 409 amino acids in *A. nidulans *and 406 amino acids in *M. grisea*. The calculated molecular weight of the protein encoded by the *sscmk1 *gene product was determined to be 45.6 kDa. This value is also in the range observed for other CaMKs in fungi, such as: 46.7 kDa in *N. crassa*, 45.6 kDa in *M. grisea *and 46.9 kDa in *A. nidulans*.

The analysis of the derived amino acid sequence revealed a calcium/calmodulin kinase containing all the 12 conserved subdomains necessary for a functional serine/threonine protein kinase [32,33], and identified a serine/threonine protein kinase catalytic domain. The nine amino acids shown to be strictly conserved in the catalytic domain of protein kinases were found in the corresponding region of SSCMK1. An additional five residues also conserved in all protein kinases were found in the corresponding region of SSCMK1. All these residues are highlighted in red in Figure 1. The PANTHER protein and gene classification system showed an extremely significant expectation value for *sscmk1 *being a calcium/calmodulin-dependent protein kinase [34].

A putative CaM-binding domain (highlighted in gray in Figure 1) was also identified in SSCMK1 using the Calmodulin Target Database search [35]. The CaM-binding domain is characterized by a high proportion of basic amino acid residues, not highly conserved at the level of primary structure, but conserved at the level of secondary structure [44]. This motif is of the same type as that found in CaM kinases of *A. nidulans *and *S. cerevisiae*, among others.

In addition to the serine protein kinase domain, the *sscmk1 *gene encoded product showed a pak-box/p21-rho-binding domain. The Rho, Rac and CDC42 are small GTPases that are involved in a variety of different fuctions such as bud emergence, actin polymerization, differentiation, protein transport and many other cellular events [45-47]. Many kinases have been shown to contain this motif and it has been reported that these protein kinases might be stimulated by the small GTPases [48]. Proteins with this domain are usually involved in the regulation of the MAPK pathway and this cascade has important relevance in the control of morphogenetic and proliferative processes.

Due to the fact that *S. schenckii *is not a genetically manageable organism, our approach to the study of the involvement of CaMK activity in dimorphism was based on the use of substances that affect CaMK activity. It is of interest to note that we used three different compounds W-7, KN-62 and lavendustin C, that will inhibit the CaMK pathway with different mechanism of action in order to lessen the uncertainties of using inhibitors that have different degrees of specificity towards CaMKs. KN-62 is the most specific inhibitor of the three, inhibiting the CaM binding site in CaMKs [49]. Lavendustin C is preferentially a CaMK inhibitor but has been reported to also inhibit tyrosine kinases [43]. Compound W-7 would be expected to inhibit CaMK and other CaM dependent proteins because it is a calmodulin inhibitor [40]. Even though there might be some considerations in terms of the specificity of these last two compounds, W-7 and lavendustin C, we have used KN-62 which is considered a very specific inhibitor of CaMKs.

Given the above, it is most important to note that the final outcomes in the presence of these inhibitors observed by us were the same regardless of the inhibitor used: 1) stimulation of the yeast to mycelium transition, and 2) inhibition of the re-entry into the yeast cell cycle. Nevertheless we have to keep in mind that the full scope of the processes inhibited by W-7 and lavendustin C are not presently known.

The inhibition observed on yeast cells induced to re-enter the budding cycle suggests that CaM and/or CaM kinase activity are required for the re-entry of yeast cells into this cycle. The controls had 50% budding cells after 9 h of incubation while those in the presence of W-7, KN-62 or lavendustin C had only 30, 28 and 38%, respectively. At 9 h of incubation the control cells undergo DNA synthesis and bud formation that results in cell duplication after 12 h of incubation [50]. The inhibition was observed at 9 h of incubation, specifically at the time of the G1/S transition. *S. schenckii yeast *cells will not form buds if DNA synthesis is inhibited [50]. We therefore believe that CaM kinase activity is probably needed for the G1/S transition during the budding cycle as has been reported for other systems as well [51].

On the other hand, these inhibitors stimulated the yeast to mycelium transition at 6 and 9 h of incubation. It is at 6 h after inoculation that control cells induced to transform to the mycelium form of the fungus first show germ tubes, after they have undergone DNA synthesis and the first nuclear division, a prerequisite for germ tube formation in this fungus [52]. It is not until 12 h after inoculation that all control cells exhibit germ tubes. The process of germ tube formation in the presence of KN-62 and lavendustin C occurred with increased kinetics, and germination was well advanced at 9 h after inoculation. Compound W-7 was observed to increase germ tube formation significantly only at 9 h after inoculation.

Two mechanisms have been postulated for the effects of CaM and CaM kinases in many systems including fungi, one suggests that these enzymes have an effect in the control of the cell cycle [51,53] and the other suggests that they have an important role in differentiation and gene expression, specifically through the activation of transcription factors [53-55]. At this moment we do not have the exact mechanism by which CaM and/or CaM kinase act in *S. schenckii*. A unified theory on how CaM kinases operate in fungi is presently not available, primarily because the targets of these enzymes are not known and contradictory results have been reported in fungi where the activity of these enzymes have been studied. In *A. nidulans*, CaMK is an essential gene, required for cell cycle progression [56,57]. In this fungus, knocking out the CaMK gene has been reported to be lethal and over-expression of the gene has been reported to be inhibitory of spore germination and growth in this fungus [56,57]. In *S. cerevisiae*, CaMK and calcineurin are not required for cell proliferation, nevertheless, they are required for survival of pheromone-induced growth arrest and for ion homeostasis [20]. In *C. albicans *CaM inhibitors were observed to suppress hyphae formation [58] while in *Paracocciodiodis brasiliensis*, CaM inhibitors were reported to inhibit the mycelium to yeast transition [59]. These results show that the role of CaMKs differ among fungal species.

Our results using CaMK inhibitors suggest that this enzyme is needed for proliferation of the yeast cells when induced to re-enter the cell cycle and is needed for the maintenance of the yeast morphology in *S. schenckii*. We could hypothesize that CaM kinase is needed for the phosphorylation of proteins involved in the regulation of the cell cycle and also for the phosphorylation and inactivation of transcription factors needed for the transition to the mycelium form of the fungus, as has been postulated for *A. nidulans *[53]. Nevertheless, the final interpretation of these results and those of the yeast to mycelium transition await the identification of the interacting partners of CaM and CaM kinases in this fungus.

## Conclusion

The information summarized in this work shows once more the complexity of the involvement of protein kinases, regulated by intracellular levels of calcium ions in the control of dimorphism in *S. schenckii*. In this work a calcium/calmodulin dependent protein kinase gene, *sscmk1*, was identified and characterized. Messenger RNA for this gene was detected in both yeast and mycelium forms of *S. schenckii*. We had previously identified a role for protein kinase C in the regulation of dimorphism in *S. schenckii *but had not identified either the presence or the role of CaMKs in this fungus. Calcium/calmodulin kinases are known to be effectors of the calcium signal in other systems but no information as to the existence and nature of these enzymes was available in *S. schenckii *until now.

The cDNA sequence of the *sscmk1 *gene revealed an ORF of 1221 bp with the predicted amino acid sequence containing all the consensus domains present in other serine/threonine protein kinases as well as a putative CaM-binding domain. The genomic sequence had five introns, whose positions are conserved in the other fungal calcium/calmodulin kinases.

The encoded protein has 407 amino acids and a molecular weight of approximately 45.6 kDa. Protein domain analysis of SSCMK1 identified the presence of a serine/threonine catalytic domain, protein kinase ATP binding region, a serine/threonine protein kinase active site, and a calmodulin binding site.

Most important of all, CaM and CaMK inhibitors were found to favor the yeast to mycelium transition and to inhibit the re-entry into the budding cycle by yeast cells in *S. schenckii *suggesting a role for calmodulin kinase in the expression of the yeast morphology of the fungus.

## Methods

### Strain

*S. schenckii *(ATCC 58251) was used for all experiments. Cultures of the mycelial and the yeast forms of this fungus were obtained as previously described [60].

### Nucleic acids isolation

DNA and RNA were obtained from *S. schenckii *yeast cells as described previously [60] using the methods of Sherman [61] and Chomczynski & Sacchi [62], respectively. Mycelium form RNA was obtained as previously described [60]. Poly A^+ ^RNA was obtained from total RNA using the mRNA Purification Kit from Amersham Biosciences (Piscataway, NJ, USA) and used as template for cDNA synthesis using the RETROscript^® ^First-Strand Synthesis Kit for RT-PCR from Ambion Corp., (Austin, TX, USA).

### Polymerase Chain Reaction

*S. schenckii *DNA (100 ng) was used as template for polymerase chain reaction (PCR) with primers (100–200 ng) targeted to conserved motifs in the *N. crassa camk-I *gene (upper primer: 5' gatgcttcccagaccatc 3'; lower primer: 5' tatgacacccattgacca 3'). The Ready-to-Go™ PCR Beads (Amersham Biosciences) were used for PCR. PCR was performed using a Perkin Elmer GeneAmp PCR System 2400 (Applied Biosystems, Foster City, CA, USA). The amplification parameters were as follows: an initial denaturation at 94°C for 30 sec, followed by 30 cycles of denaturation step at 94°C for 30 sec, annealing at 40–55°C (depending on the primer combination) for 1 min, and extension at 72°C for 2 min.

PCR products were analyzed on agarose gels and the DNA recovered using Spin-X Centrifuge Tube Filters as described by the manufacturer (0.22 μm, Corning Costar Corp., Corning, NJ, USA). The TOPO TA Cloning System (Invitrogen Corp., Carlsbad, CA, USA) was used for cloning PCR products and One shot™ *E. coli *(TOPO 10) competent cells were transformed with the ligated products. Plasmid preparations were obtained using the Fast Plasmid TM Mini technology from Eppendorf (Brinkmann Instruments, Inc. Westbury, NY, USA).

### Rapid amplification of cDNA ends (RACE)

The 5' and 3' ends of the *S. schenckii sscmk1 *gene were obtained using the SMART RACE Kit (BD Biosciences Clontech, Palo Alto CA, USA). Primers for RACE were designed based on the sequence obtained previously. The gene specific primers (GSP) and nested gene specific primers (NGSP) for the 5' touchdown and nested reactions were the following: 5' RACE: 5'GSP1-1, 5' cggagtcagactgctttgtaaggtacag 3'; 5'GSP1-2, 5' cgattcaaaccagtccacaaagcggaca 3'; 5'NGSP1-1, 5' tgtaaggtacagaaggttttccggtttt 3'; 5'NGSP1-2, 5' ttacccttgacattcttattcaggatga 3'. The gene specific primers (GSP) and nested gene specific primers (NGSP) for the 3' touchdown and nested reactions were the following: 3'GSP2-1, 5' ccttctgtaccttacaaagcagtctgac 3'; 3'GSP2-2, 5' ttccgttccgaaaacctgcccgacctga 3'; 3'NGSP2-1, 5' aagcagtctgactccgacttggttttgg 3'; 3'NGSP2-2, 5' tgccactgaccacaacttgctgccagag 3'. All RACE reactions were carried out in the Perkin Elmer GeneAmp PCR System 2400 (Applied Biosystems). The touchdown PCR and nested PCR parameters were the same as those used previously [60].

### cDNA and genomic sequence

In order to obtain the complete cDNA coding sequence for *sscmk1*, PCR was performed using cDNA as template (100 ng) and the *sscmk1 *forward primer 1 (5' ccccattcaccctctccc 3') and *sscmk1 *reverse primer 1 (5' ccacaagagcaaacagcc 3'). To obtain the genomic sequence, PCR was performed using DNA as template and the *sscmk1 *forward primer 2 (5' accaactccaatatgagc 3') and *sscmk1 *reverse primer 1 above. The PCR products containing the entire coding sequence, from both the cDNA and genomic templates were cloned and sequenced.

### DNA sequencing and analysis

All sequencing reactions were conducted using the ABI PRISM™ 377 automated DNA sequencer (Applied Biosystems) and the Thermo Sequenase II Dye terminator Cycle Sequencing Premix Kit (Amersham Biosciences) as described previously [60].

### Bioinformatics Sequence and Phylogenetic Analysis

The theoretical molecular weight was calculated using the online ExPASy tools [63]. The partial 5' UTR nucleotide sequence of *sscmk1 *obtained by RACE was analyzed for transcription factor binding sites using online MatInspector [28]. On-line ScanProsite search [30] and Pfam [31] were used to identify potential motifs present in SSCMK1. The calmodulin-binding domain was identified using the on line Calmodulin Target Database [35]. The protein classification was performed using the PANTHER Gene and Protein Classification System [34].

On-line database searches and comparisons were performed with the BLAST algorithm [64] with a cutoff of 10^-7^, a low complexity filter and the BLOSUM 62 matrix. A multiple sequence alignment was built using MCOFFEE [65]. The alignment was visualized using the program GeneDoc [66]. The alignment was analyzed for conservation using the program G-blocks [67] and trimmed as suggested by this software. The trimmed alignment was used to build a phylogenetic tree using the PHYLIP suite of programs [68] with 1000 bootstrap replicates, using the neighbor-joining algorithm and an extended majority rule for consensus.

The multiple sequence alignment and the phylogenetic tree for CaMKs were constructed using the following sequences of either CaMK or hypothetical proteins homologous to CaMKs: *Coccidiodes immitis *(GenBank: EAS33607.1);*Emericella nidulans *(GenBank: AAB97502.1); *Gibberella zeae *(GenBank: EAA76718.1); *Kluyveromyces lactis *(GenBank: CAG98820.1); *Magnaporthe grisea *(GenBank: XP_365067.1); *Metarhizium anisopliae *(GenBank: AAB80685.1); *Neurospora crassa *(GenBank: AAL14118.1); *Phaeosphaeria nodorum *(GenBank: ABD59786.1); *Yarrowia lipolytica *(GenBank: CAG80077.1); *Saccharomyces cerevisiae *(GenBank: CAA40928.1) and *Homo sapiens *CaMK1D (GenBank: AAH35745.1). The GenBank accession numbers for the *S. schenckii *calcium/calmodulin kinase are the following: mRNA, AY823267; genomic sequence, AY823266; and derived amino acid sequence, AAV80434.

### Inhibitor studies during the yeast to mycelium transition and the yeast cell cycle

In order to evaluate the involvement of CaM and CaMK in the control of dimorphism in *S. schenckii*, inhibitors were added to the medium where synchronized yeast cells were induced to undergo transition to the mycelium form or re-enter the budding cycle. The following substances were tested for their effects on the yeast to mycelium transition and the yeast cell cycle: compound W-7, [*N*-(6-aminohexyl)-5-chloro-1-naphthalene-sulfonamide] (100 μM) [38], KN-62, 1- [*N*,*O*-bis(1,5-isoquinoline-sulfonyl)-*N*-metyl-1-tyrosyl]-4-piperazine (10 μM) [41] and lavendustin C (10 μM) [43] (Calbiochem, EMD Biosciences Inc.).

The yeast form of the fungus was obtained from conidia as described previously [52]. Unbudded yeast cells were synchronized by selection of unbudded cells by filtration [52]. These cells were induced to form germ tubes in the presence and absence of effectors of CaM and CaM kinase activity in a basal medium with glucose at pH 4.0 [52]. Parallel cultures were inoculated and, at 6 and 9 h after inoculation, the content of a flask was filtered for the determination of the percentage of cells with germ tubes for each of the substances tested. These same yeast cells were induced to re-enter the yeast cell cycle as described previously in the presence and absence of effectors of CaM or CaM kinases in a basal medium with glucose at pH 7.2 [50]. Yeast cells were inoculated as described previously [50] and at 6 and 9 h after inoculation samples were taken and the percentage of budding cells was recorded.

The results are expressed as the average percentage of cells with germ tubes or buds at 6 and 9 h of incubation ± one standard deviation of at least three independent determinations. The Student t test was used to determine the statistical significance of the data. A 95% confidence level was used to determine statistical significance.

## Authors' contributions

LVA carried out all the molecular biology studies, gene cloning and identification of *sscmk1 *gene and also drafted parts of the manuscript. SVB contributed with the inhibition studies. RGM carried out the sequence alignments, domain characterization and phylogenetic tree. NRV designed the study, drafted the manuscript, participated in sequence alignments and domain characterization and conducted the inhibition studies.

All authors have read and approved the final manuscript.
